# Similarity-Based Adaptive Window for Improving Classification of Epileptic Seizures with Imbalance EEG Data Stream

**DOI:** 10.3390/e24111641

**Published:** 2022-11-11

**Authors:** Hayder K. Fatlawi, Attila Kiss

**Affiliations:** 1 Department of Information Systems, ELTE Eötvös Loránd University, 1117 Budapest, Hungary; 2 Center of Information Technology Research and Development, University of Kufa, Najaf 540011, Iraq; 3 Department of Informatics, J. Selye University, 94501 Komárno, Slovakia

**Keywords:** machine learning, similarity analysis, EEG, imbalanced data

## Abstract

Data stream mining techniques have recently received increasing research interest, especially in medical data classification. An unbalanced representation of the classification’s targets in these data is a common challenge because classification techniques are biased toward the major class. Many methods have attempted to address this problem but have been exaggeratedly biased toward the minor class. In this work, we propose a method for balancing the presence of the minor class within the current window of the data stream while preserving the data’s original majority as much as possible. The proposed method utilized similarity analysis for selecting specific instances from the previous window. This group of minor-class was then added to the current window’s instances. Implementing the proposed method using the Siena dataset showed promising results compared to the Skew ensemble method and some other research methods.

## 1. Introduction

**Figure 1:**
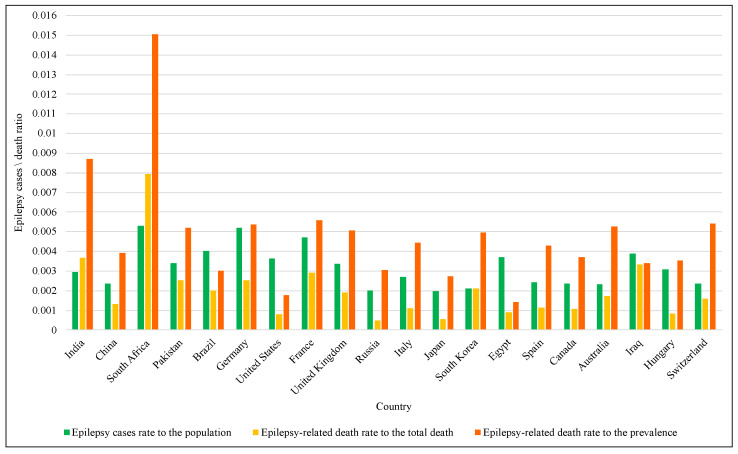
The rates of Epilepsy cases and related deaths.

The treatment of chronic diseases, including epilepsy, and the care of patients, is of great importance in health systems worldwide. Epilepsy is one of the most prevalent chronic neurological diseases, as the World Health Organization confirms that 50 million people around the world suffer from seizures to different degrees. Those with epilepsy have three times the risk of premature death than those without it [1]. The number of fatalities and impairments brought on by this condition in the U.S. climbed from 0.3 per 100,000 people in 1999 to 0.5 in 2019 [2]. Figure 1 illustrates the rates of epilepsy-related death and epilepsy cases in 20 countries around the world. It can be seen that in 16 of them, the death rate to the prevalence was more significant than the epilepsy cases rate to the population.

The activity of the human brain’s neurons generates a sequence of non-stationary and random electric signals. Beneficial information can be obtained from collecting these signals to facilitate monitoring of the brain’s health [3,4,5]. This activity can be recorded using the electroencephalogram (EEG) technique by invasive or non-invasive electrodes on the human scalp [6,7,8]. Classifying EEG signals for seizure detection requires some pre-processing steps to remove noise and feature extraction. The most valuable information is extracted from the signals as tabular data using many techniques such as Fast Fourier Transformation (FFT) [9].

Classifying stream data depends on the principle of adapting the drift in the distribution of data through a partial change in the classifier structure [10]. The adaptation process can produce a more stable model with a balanced-class data stream, which means that if the task is a binary-class classification, the weight (number of occurrences) for one class is close to the other’s weight. On the contrary, if the stream is unbalanced, meaning that one of the classes appears more than the other, the model will be biased towards the most frequent class [11,12]. This model produces an unrealistically high accuracy that depends on the dominance of the major class. In many classification tasks, including the classification of an epileptic seizure, the rare class is the most important. The classification model is ineffective when seizure signals are incorrectly classified as normal signals because of the unreliable structure of the classifier.

Most methods that deal with unbalanced data streams depend on utilizing a set of classifiers, i.e., Ensemble model, minor class oversampling, and major class undersampling. Despite the improvement of positive class detection by using undersampling to reduce the dominance of negative class, it will produce an increase in false positive classification. In addition, accumulating the elements of the positive class with window size during the stream processing may change the majority from the negative class to the positive class. Because most EEG signals are normal, making many misclassifications of these normal signals as epilepsy seizures will lead to a waste of medical efforts and a lack of reliability in the classifier. This work aims to contribute by avoiding these problems as follows:

- Preserving the classifier’s ability to detect normal EEG signals by keeping all the data elements from the negative class without undersampling, thereby reducing false positive alarms.
- Improving the classifier effectiveness for detecting the seizure signals by strengthening the presence of positive class within the current window using elements from the previous window. These items are selected based on their similarity to the current window’s items and their recency.
- Reducing the required computational resources for the proposed method by choosing the center of the current window as a representative point for calculating the similarity instead of all window items.

## 2. Literature Review

Despite their effectiveness in improving the accuracy of classifying the positive class, most current methods accomplish this at the expense of classifying the negative class. Using undersampling for the negative class elements with a fixed window size may significantly change the data’s original distribution. In contrast, accumulating positive data elements with an adaptive window size may increase the computational resources required to modify the current classification model. Both situations can produce an unreliable classification model. In this section, a number of the basics of unbalanced data, adaptive classification, and similarity analysis will be explained, as well as a review of several research-related works.

### 2.1. Imbalance EEG Data Stream

The phenomena of imbalance in classes (i.e., the targets of classification tasks) can be seen in plenty of datasets, especially those related to diseases. Mostly in this type of dataset, the number of disease-positive data records is remarkably less than the number of normal data records. This issue can decrease the classifier’s effectiveness because it relies on accuracy measures that ignore the rare appearance of the minority class [13]. Many techniques are proposed to handle this issue by modifying the classifier to focus more on the minority, called the Algorithm level, or by adjusting the data itself, called the Data level. In the latter type, undersampling of major class and/or oversampling of minor class can be applied [13]. Many techniques, such as Skew Ensemble Algorithm [11,14,15], have been developed for handling the unbalanced class distribution in data streams. It depends on preserving the positive examples of the previous data chunks. It also includes reducing the number of negative examples by applying random undersampling. Our work includes Data level processing by minor class oversampling.

According to [16], the average range for the duration of different seizure types is between 18 and 130 seconds. This period is significantly short compared to the time when there is no epileptic seizure; therefore, EEG data can be considered clearly unbalanced data. The epileptic seizure data class in this work will be referred to as a positive, minor, or rare class, while the normal data class will be referred to as a negative or major class. Important information can be extracted from EEG signals in the features extraction process using Fourier transform (FT). As EEG is a periodic signal, Discrete Fourier Transform (DFT) converts it to the frequency domain. Let the signal be $h_{r}$ for r=0…R−1 and $h_{r}$ = $h_{r+q_{R}}$ for all *r* and *q*. The discrete Fourier transform of *h* is [17]:

(1) $$H_{k}=\sum_{0}^{R-1}e^{-i\frac{2\pi }{r}kr}h_{r}$$

### 2.2. Adaptive Classification

Classification of data streams requires a method for detecting and responding to a change in data distribution. This change (which is usually referred to as concept drift) could cause a modification to the current structure of the classification model [18]. Adaptive sliding window (ADWIN) is a popular tracking method with an adaptive size that changes in response to the change in the average of items [10]. As a result of the features of the decision tree classifier, many versions have been developed to deal with data streams such as Very Fast Decision Tree (VFDT) or Hoeffding Tree. This classifier is characterized by its ability to respond to a change in the distribution of data by changing the leaf nodes in the tree into decision nodes (i.e., that split data) using specific statistical data. VFDT is used as a base learner in many ensemble classifiers, such as Adaptive Random Forest (ARF) [10,19,20].

### 2.3. Similarity Analysis

Research interest in the field of similarity analysis continues in many areas such as Clustering [21,22,23,24], Classification [25,26,27,28], and Recommendation systems [29,30,31,32,33]. In data stream analysis, Similarity has many recent research articles as well [34,35,36,37,38,39]. Generally, similarity analysis is concerned with finding similar data points by measuring the distance between them for the purpose of exploring specific patterns. A similarity distance *d* between two data points *a* and *b* is a positive number with a range between 0 and 1. The more different the two points are, the closer the value of *d* is to zero [40,41]. Euclidean distance is one of the most popular measures used with numerical attributes. If *a* and *b* have multiple attributes, Euclidean distance d(a,b) can be calculated as follows [41]:

(2) $$d\left(a,b\right)=\sqrt{\sum_{i=1}^{m}{\left(a_{i}-b_{i}\right)}^{2}}$$

In many machine learning tasks, it is more practical to calculate a distance to a representative data point than to all data points. The centroid point is one of the most common representers, and it can be calculated using the arithmetic mean of feature values of a specific data subset. Let *X* be a numeric feature with *N* values; the mean of *X* is [41]:

(3) $$\hat{X}=\frac{\sum_{i=1}^{N}xi}{N}$$

### 2.4. Evaluation of Classification Performance

The classification performance can be evaluated using many measures, such as Sensitivity, Specificity, Accuracy, Precision, and F1-score. Calculating those five measures’ values depends on a basic table called the confusion matrix. In binary classification tasks, there are only two possible values for the target of the classification process. The first one is the Negative class, which refers to the normal value in the given data, i.e., normal EEG signal, and it mostly represents the majority value. The second one is the Positive class which refers to the abnormal status, i.e., seizure EEG signal. For this type of classification, the confusion matrix has two rows that refer to the actual values and two columns for the values predicted by the classifier. True Positive (TP The format of the letters mentioned in the equation should be unified.) denotes the number of positive instances that are correctly classified as positive. In contrast, True Negative (TN) refers to the number of negative instances that are correctly classified as negative. False Negative (FN) is the number of positive instances incorrectly classified as a negative; also, False Positive (FP) refers to the number of negative instances incorrectly classified as a positive. True Positive Rate (TPR) (i.e., Sensitivity), True Negative Rate (TNR) (i.e., Specificity), False Positive Rate (FPR), and False Negative Rate (FNR) can be calculated as follows:

(4) $$TPR=\frac{TP}{TP+FN}$$

(5) $$TNR=\frac{TN}{TN+FP}$$

(6) $$FPR=\frac{FP}{FP+TN}$$

(7) $$FNR=\frac{FN}{FN+TP}$$

On the other hand, Accuracy, Precision, and F1-score can be calculated as follows:

(8) $$Accuracy=\frac{TP+TN}{TP+TN+FP+FN}$$

(9) $$Precision=\frac{TP}{TP+FP}$$

(10) $$F1-score=\frac{2TP}{2TP+FP+FN}$$

### 2.5. Related Works

Gradual Resampling Ensemble (GRE) was proposed by [42] to provide an ensemble classifier with the ability to deal with an imbalanced data stream. Their method utilized the clustering technique DBSCAN to choose a part of minority data examples and combine them with the current set. These examples are selected on the basis of the weakness of the likelihood of overlapping with the current set. Their results showed a better performance than many techniques by preserving the performance of the minority and majority classes.

The problem of class imbalance has been dealt with implicitly in [43] using under-sampling in the imperceptible classes’ evolution. Their method presented an ensemble classifier that provides a single learner for each class, then adapted it dynamically for the new data. Their results showed a good performance in terms of adaptation and efficiency in responding to the class distribution changes, including emergence of the new class, the disappearance of a current one, and the recurrence of an old class.

The Geometric Deep Learning method is proposed in [44] as a subject-independent classifier for predicting the onset of an epileptic seizure. Their approach tried to overcome the limitation of training data in some subjects. The grid connection of EEG signals was utilized in their method to derive the graphs. The evaluation of their method using two real datasets, CHB-MIT and Siena, showed comparable results with state-of-the-art methods for predicting the seizure. A combination of electroencephalogram (EEG) and electrocardiogram (ECG) signals data was utilized by [45] for predicting seizure onset. The classification process in their model was performed by a support vector machine (SVM) that was applied to synchronized features of EEG signals.

To avoid inefficient consumption of memory in classifying an imbalanced data stream, ref. [46] proposed a chunk-based ensemble technique. The proposed method depends on the current chunk with more focus on misclassified data items and does not need the previous data. Their results showed good effectiveness compared with some other techniques; however, in terms of efficiency, the performance was not the best. Another ensemble algorithm was proposed by [12] to reduce the effect of imbalanced class distribution. Their method was based on cost-sensitivity, and automatically calculated the cost of misclassified data samples. Detection of intrusion behavior in SDN networks was chosen as an application for the proposed algorithm.

While most of the research works in the imbalance class field focus on a binary class task, ref. [47] proposed a generalized model for the multi-class task. They utilized a Gaussian kernel function with an extreme learning machine and class proportion for choosing the regularization parameters. A label-noise-tolerant classifier has been proposed by [48] as an improvement for the Synthetic Minority Oversampling Technique (SMOTE). It overcomes the drawback of extra parameters in SMOTE using the self-adaptive method. Their method utilized relative density as a measurement of minority-class samples, and their results confirm their preference. An undersampling mechanism has been proposed by [49] to choose specific instances from the majority class using a set of density peaks. Their method included an automatic detection of the undersampling size by weighting each instance.

## 3. Methodology

The proposed method aims to increase the presence of positive-class instances in the current window of the data stream by using some previous instances. These old instances must be close (i.e., similar) to the new ones in the current window, and their presence time does not exceed a certain threshold. Initially, the proposed method receives EEG signals from wearable sensors and applies preprocessing to extract important features using FFT. Those features will be used as the input for a similarity-based adaptive window. The resulting data in this adaptive window will be utilized to adapt the current ensemble classifier of ARF. In this section, the mechanism of the proposed method will be explained within the classification model for epilepsy detection using ARF.

### 3.1. EEG Stream Preprocesing

This step begins by collecting EEG signals from the brain of an epilepsy patient using physical sensors. These sensors generate a vast number of data instances; therefore, some preprocessing operations are needed with these produced data before using them in the classifier. This step includes applying one of the implementations of the Discrete Fourier Transform (explained in Section 2.1), i.e., Fast Fourier Transform (FFT). Despite the computational cost of this operation, FFT will highlight the most valuable features in the signals, thereby improving the quality of the construction of the classifier.

### 3.2. Similarity-Based Adaptive Window (SAW)

Similarity-based Adaptive Window (SAW) represents a new layer between the preprocessing steps and the ARF classifier. Let St be the set of preprocessed instances that newly arrive in time moment T. The subset of elements in St with a positive class denotes P(T), while N(T) refers to elements with a negative class. The Positive Instance Age (PIA) timer for all Pt elements will be set to 0. The centroid C(T) of Pt will be calculated as a midpoint for all features of P(T) based on the Equation (3), and as a result of this calculation, C(T) can be a real data point (i.e., the values of C(T) match one of the data instances in the current window) or a virtual point representing the mean of data instances.

**Figure 2:**
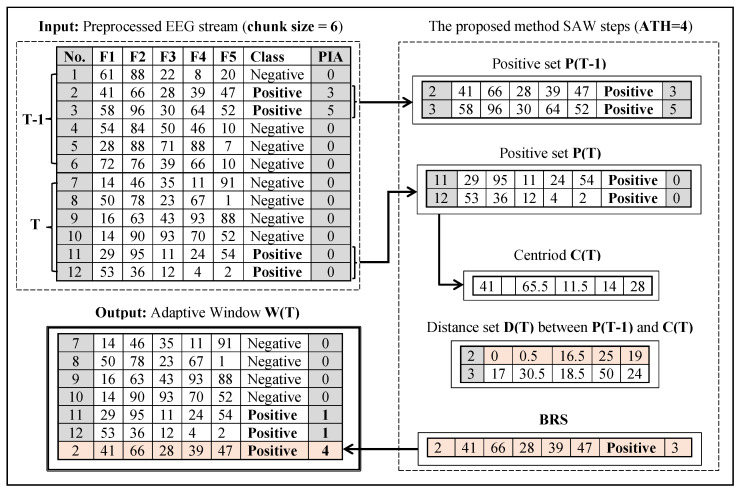
Detailed example for the proposed method SAW steps.

SAW preserves a subset of positive instances from the previous time moment T-1 to merge them with the current instances in time T. It works like a balance set to prevent the model from ignoring the rare (i.e., positive) class because of the dominance of the major class, which is the case in the EEG data stream. A Balance Rare-class Set (BRS) is a subset from P(T-1) that has a shorter distance from the centroid C(T). Euclidean distance will be used to calculate the distance based on Equation (2). The size of BRS will be controlled by using another constraint, the PIA threshold. Each BRS element should have PIA value less than a specific value defined by the user. Algorithm 1 and Figure 2 explain the steps of the proposed method SAW.
**Algorithm 1** SAW for imbalanced EEG.**Input:** EEG preprocessed data stream S, ATH ← Threshold of the maximum PIA1:**for** each time T **do**2:    P(T-1) = positive-class instances in S(T-1)3:    S(T) = Preprocessed EEG stream instances in time T4:    P(T) = positive-class instances in S(T)5:    Set PIA counter of P(T) elements to 06:    **if** (Length(P(T)) != 0) AND (Length(P(T-1)) != 0) **then**7:        Find centriod C(T) of P(T)                                         ▹ based on Equation (3)8:        Compute distances set D of P(T-1) from C(T)         ▹ based on Equation (2)9:        Sort D in ascending order10:      BRS = top elements of D that have PIA < ATH11:      Increase PIA of BRS elements by 112:      W(T) = Merge S(T) with BRS13:    **end if**14:**end for**15:**Return** Adapted Window W(T)

According to the number of the positive instances in the current and previous window, SAW has four cases:

- Both current and previous windows have no positive instances (i.e., seizure signals), in this case the next steps of SAW will not be applied.
- Previous window does not have positive instances, then SAW next steps will not be applied and the current window instances will stay the same without any modification. As SAW is an accumulative procedure, this case can occur rarely.
- Current window does not have positive instances, then BRS will be formed directly from P(T-1) elements that have PIA < ATH.
- Both current and previous windows have positive instances, in this case SAW steps will be applied as in Algorithm 1.

Figure 2 describes SAW steps using 12 synthesized data points that have been chosen randomly in the range 1 to 100. The example contains two windows with 6 instances for each, and ATH parameter set to 4. The figure shows that size of window T has changed in the end of SAW from 6 to 7 by adding the positive instance No. 2 that belonged to window T-1.

### 3.3. ARF

ARF ensemble classifier was proposed by [50] as a development of the original random forest to classify a data stream. It contains three major parts:

- A base learner: the classification technique used to classify the targeted data. In this work, VFDT is utilized as a base learner. The ensemble size (i.e., number of base learners) and the size of the decision tree (i.e., number of terminal nodes) are user-defined parameters that affect the building ensemble model process.
- Resampling method: specify how to choose the data subset for each base learner. ARF utilizes online bagging that uses Poisson(1) distribution for this task.
- ADWIN for drift detection method. It has two parameters: Warning threshold and Drift threshold. Their task is to detect when ARF built a background tree and replace the current tree with it.

ARF receives the adaptive window stream as the output of SAW, then divide it into M subsets according to online bagging, where M is equal to the ensemble size. Each subset will be used to adapt a specific VFDT. Building VFDT is an iterative procedure; the best feature is chosen for splitting the current data in each iteration. The evaluation of a feature depends on the impurity of class distribution before and after the splitting process. Information Gain of feature Fr represents the difference between the entropy of the parent node and the entropy of the child node. After using Fr, entropy of node *R* could be computed as follows:

(11) $$Entropy\left(R\right)=-\sum_{i=0}^{cl-1}p\left(i|R\right)log_{2}p\left(i|R\right),$$

where cl denoted the number of classes.

**Figure 3:**
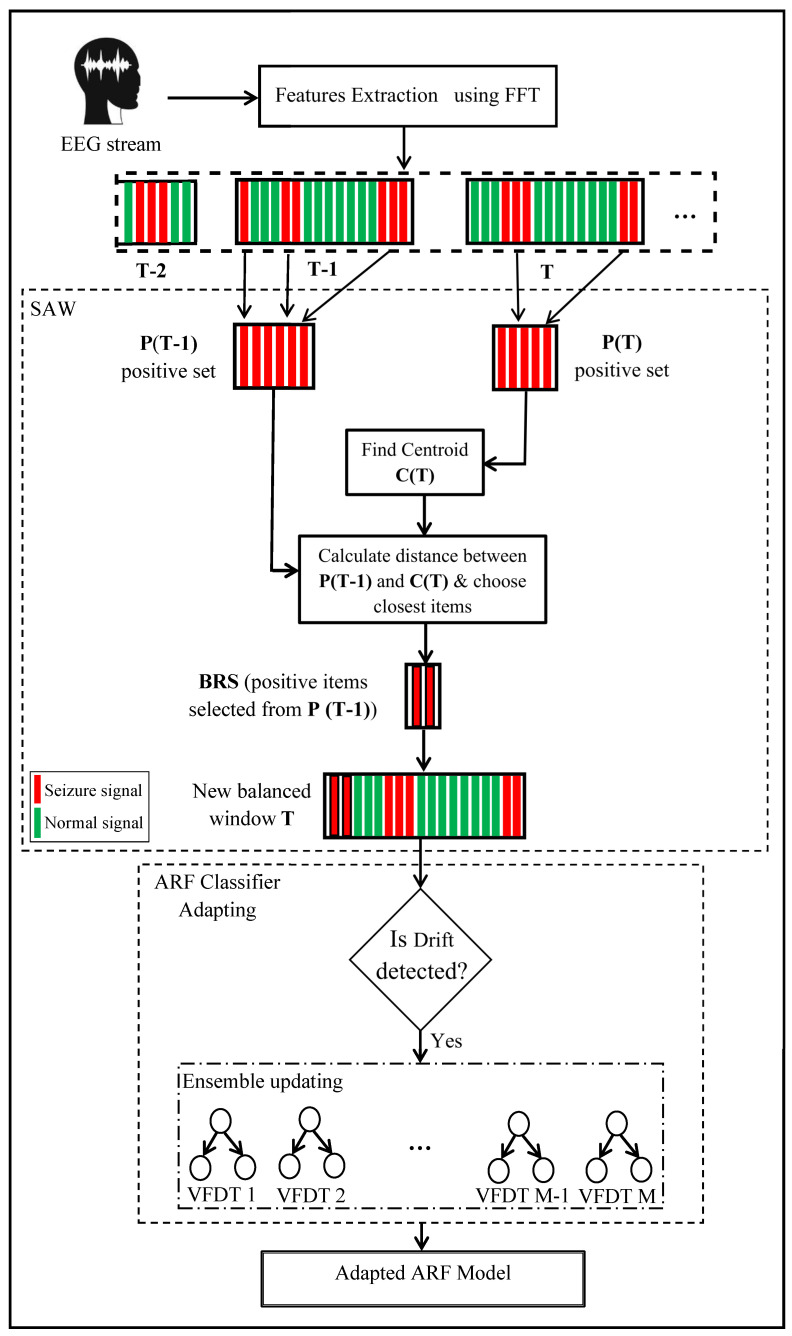
Diagram of EEG stream classification including preprocessing, Similarity-based Adaptive Window, and ARF classifier adaptation.

All steps of the three layers; preprocessing using FFT, the proposed method SAW, and adapting the ARF ensemble classifier; are illustrated in Figure 3.

## 4. Implementation and Results

**Figure 4:**
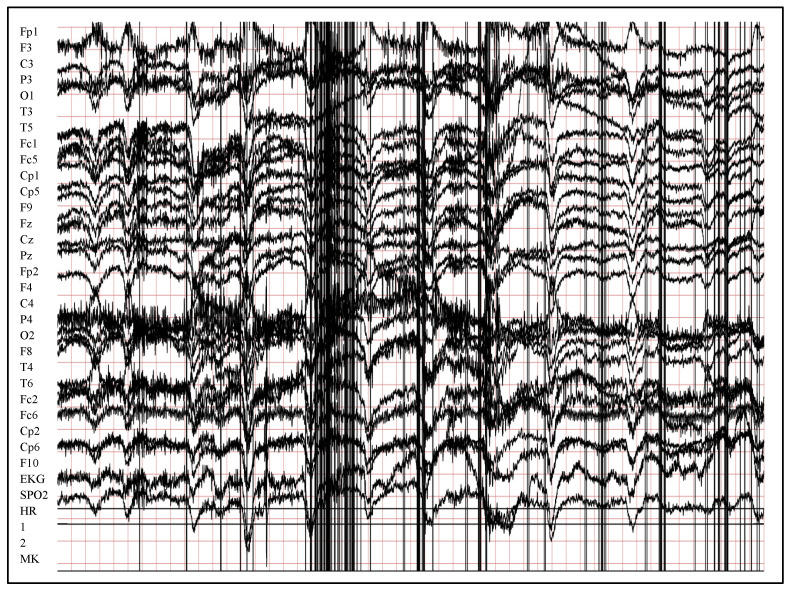
Illustration of 10 s normal EEG signals from raw Sienna data using LightWAVE.

**Figure 5:**
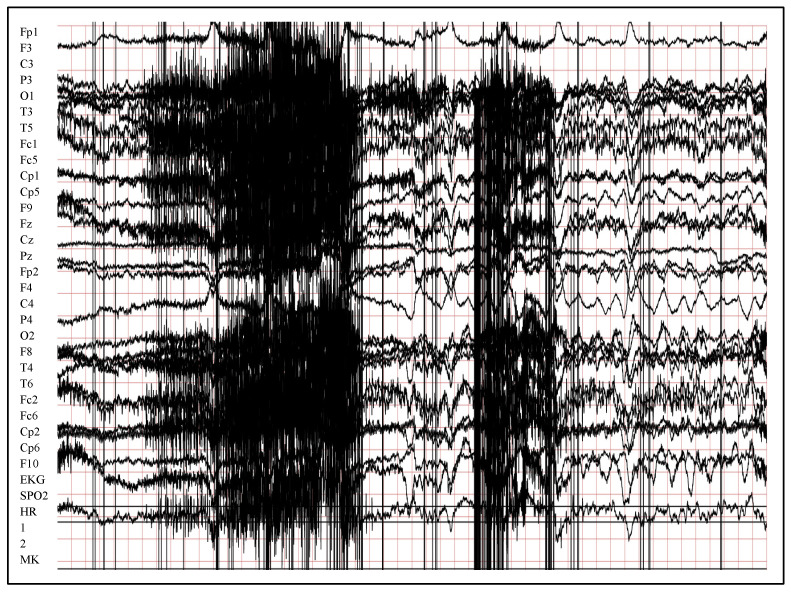
Illustration of 10 s seizure EEG signals from raw Sienna data using LightWAVE.

The verification of the proposed method has been performed using a real, new, and huge dataset called Siena Scalp EEG Dataset [51,52]. It contains more than 20 GB of EEG signals that were gathered from 14 patients at the University of Siena, Italy. In addition to its large size, the Siena dataset is distinguished by the fact that the data are taken from a wide age range, between 25 and 71 years for nine male patients, and between 20 to 58 years for five female patients. Figure 4 illustrates a sample of 10 seconds of normal EEG signals, while Figure 5 illustrates the same duration of seizure EEG signals. The signals in both Figure 4 and Figure 5 were chosen from the first patients’ files and have been illustrated using LightWAVE [53]. Due to the limitation of available computation resources in our implementation, 22 files from all 14 patients have been selected to collect signals of 164,291 s. One file has been selected from each patient that has single or multiple large file(s), while many files have been chosen randomly from patients with many medium files. This is excepting the first patient’s, all of whose files have been used because the file size was small. Table 1 shows the subset of files used from Siena Scalp EEG Dataset, and the unbalanced distribution between the number of received normal signals (162,987) and seizure signals (1304).

### 4.1. The Implementation Platform

This implementation utilized two essential Python libraries: PyEDFlib and scikit-multiflow. As EEG data are stored in European Data Format EDF files, PyEDFlib was used for reading EEG signals, features extraction using FFT, and data row aggregation. The University of Waikato’s ECOSYSTEM project developed a scikit-multiflow open-source package for data-stream mining tasks and stream generators. Data streaming and stream classifiers have been performed in this implementation using this package. A Windows PC with an Intel Core i7 8-core processor and 16 GB of memory, with Anaconda 2.1 and Spyder 5.1 used to execute Python code.

### 4.2. Experiment Results

**Figure 6:**
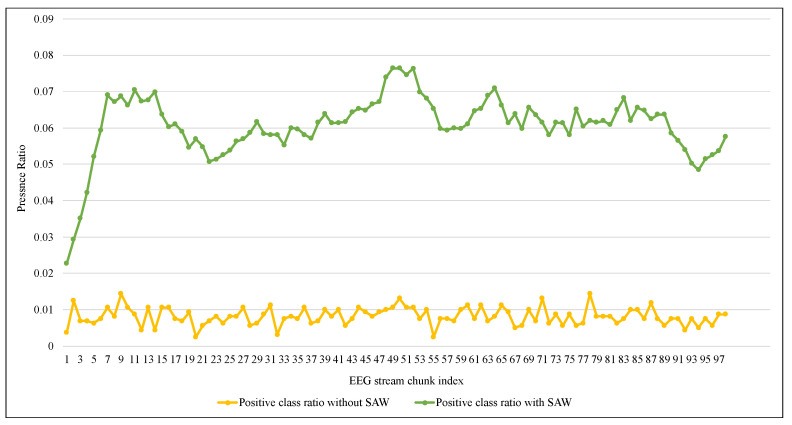
The ratio presence of minor class in the stream with and without SAW.

In order to enhance the presence of data elements of the positive class, the proposed method was applied to the Sienna dataset after dividing its data equally into 100 chunks. Each chunk represents a window with an initial size of 1600 data instances. This process aimed to simulate the flow of the EEG data stream. In Figure 6, we can notice that the presence of the positive class was mainly less than 0.01% before applying the proposed method, while it gradually increased to between 0.06% and 0.07% after its application.

The performance improvement of ARF has been shown in (Table 2 and Table 3), which compare the classification quality of the original ARF, which does not handle an imbalanced data stream and utilizes the Skew algorithm, and the proposed SAW method. The comparison included seven measures, TPR, TNR, FPR, FNR, Accuracy, Precision, and F1-score, which have been mentioned in Section 2.4. SAW performance has also been evaluated using another three stream classifiers, as shown in Table 4. Those classifiers were a single model of VFDT, Extreme Fast Decision Tree (EFDT), and K-Nearest Neighbor (K-NN). This comparison showed that SAW had its best results with the ARF classifier. The implementation of the Skew ensemble algorithm has been performed for evaluation purposes, and it was limited to the imbalance stream handling part, as ARF has its ensemble strategy.

**Figure 7:**
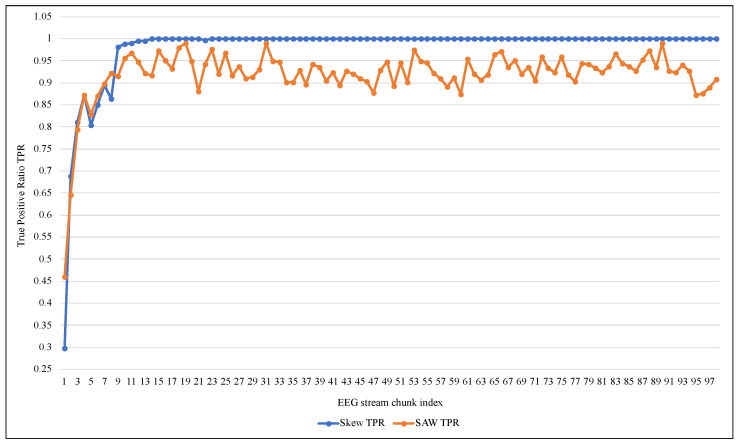
Performance comparison of ARF with and without SAW using True Positive Rate.

**Figure 8:**
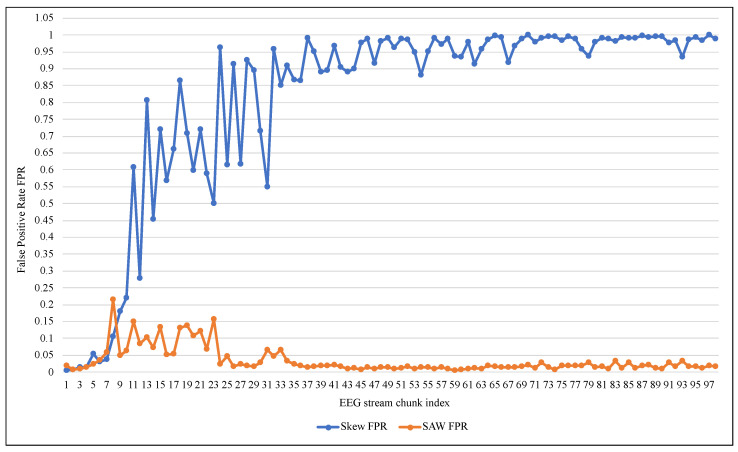
Performance comparison of ARF with and without SAW using False Positive Rate.

**Figure 9:**
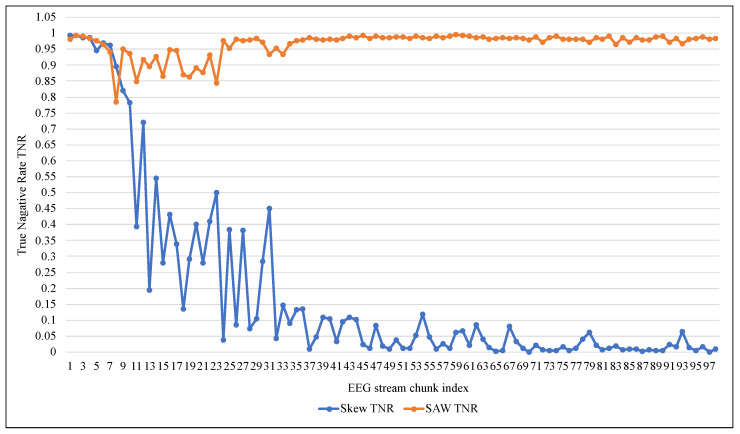
Performance comparison of ARF with and without SAW using True Negative Rate.

**Figure 10:**
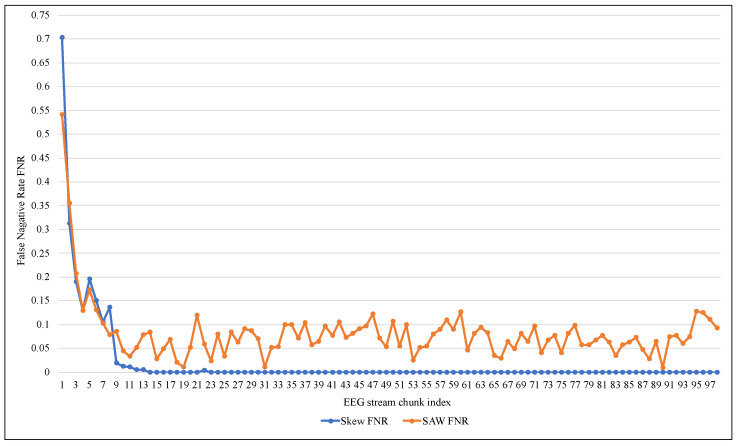
Performance comparison of ARF with and without SAW using False Negative Rate.

Table 3 shows that despite the high accuracy and TNR values of 99%, ARF cannot deal with an unbalanced EEG data stream as it had a very low TPR, reaching 0.006 on average. ARF tended to classify the new data elements as belonging to the negative class because of its majority. Since the Skew ensemble algorithm that was explained in Section 2.1 works to preserve all the data elements of the positive class, the value of TPR was higher compared with SWA, as shown in Figure 7. However, this can not be referred to as a good performance because Skew resulted in a high FPR. In contrast, the proposed method SAW resulted in a significantly low FPR, reaching less than 0.033, as seen in Figure 8. On the other hand, a comparison between the two methods in terms of TNR showed that the performance of the Skew ensemble gradually declines until it reaches a low level of 0.05, while the performance of SAW improves significantly to achieve an outstanding level after 30 data chunks, as shown in Figure 9. In Figure 10, we can see that both methods had a good value for FNR. It should be noted that the Skew ensemble had lower FNR because of its excessive tendency toward the positive class.

**Figure 11:**
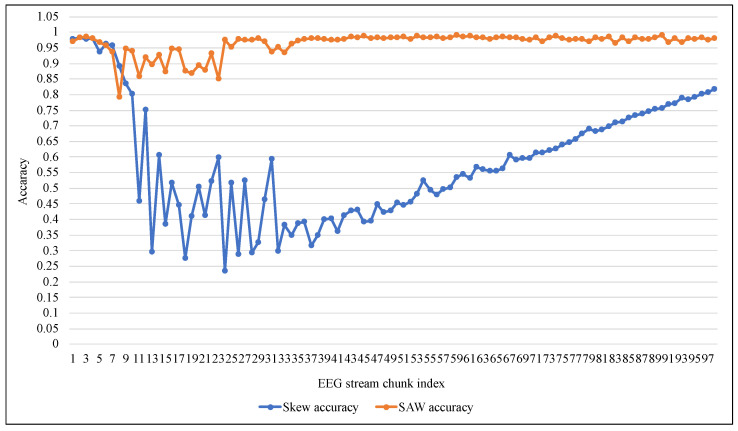
Performance comparison of ARF with and without SAW using Accuracy.

**Figure 12:**
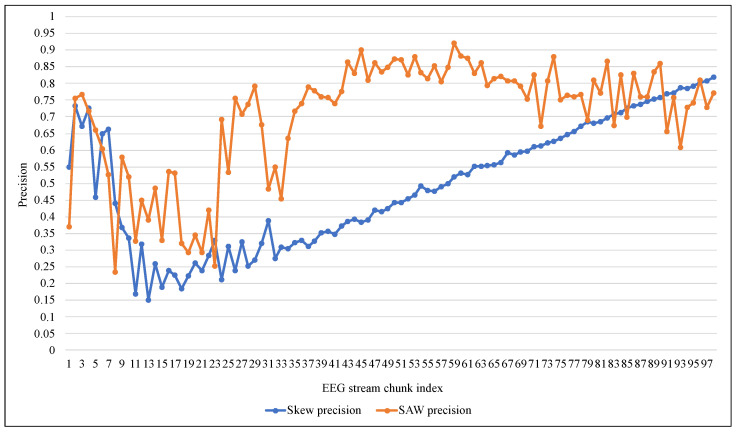
Performance comparison of ARF with and without SAW using Precision.

Figure 11 and Figure 12 summarize the tracking of ARF performance using both methods in the terms of Accuracy and Precision. The accuracy confirmed the proposed method’s superiority and stability against the low and unstable performance of the original method. Despite the poor performance of both methods at the beginning of the training process, according to the precision metric SAW improved to about 80% on average after 40 data chunks. At the same time, Skew needed 95 chunks to reach that as shown in Figure 12. It should also be noted that the significant changes in the value of the measures (i.e., improvement of TPR or decay of FNR) in Figure 7, Figure 8, Figure 9, Figure 10, Figure 11 and Figure 12 at the beginning of training were due to the increased presence of positive class instances. These instances at the beginning of SAW training were very limited and then began to rise until they reached a stable number after approximately nine chunks, depending on the value of PIA.

**Figure 13:**
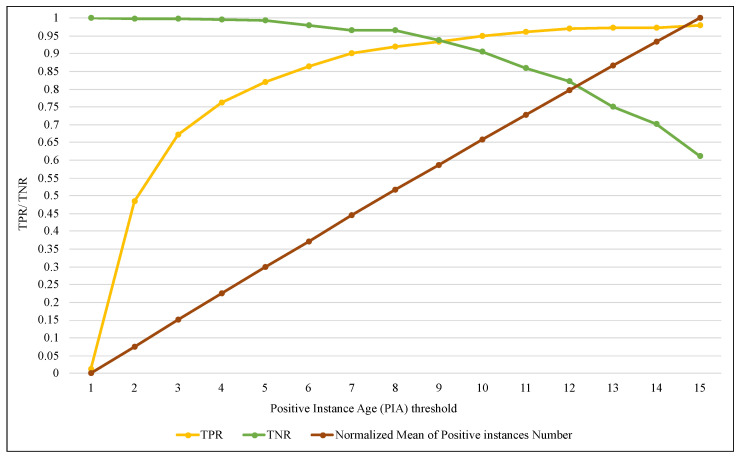
Tracking the performance of ARF using TPR and TNR with different ranges of PIA threshold.

**Figure 14:**
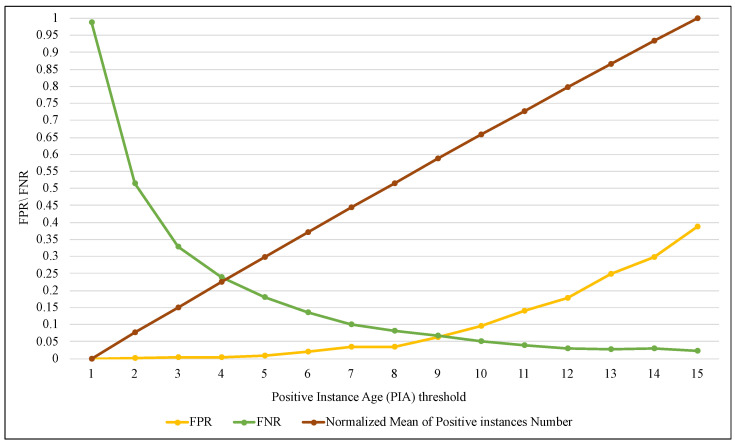
Tracking the performance of ARF using FPR and FNR with different ranges of PIA threshold.

**Figure 15:**
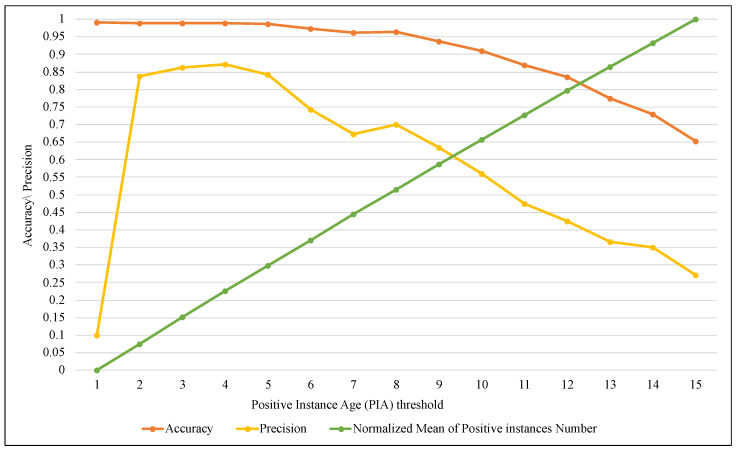
Tracking the performance of ARF using Accuracy and Precision with different ranges of PIA threshold.

BRS is one of the essential components of the proposed method because it allows keeping a limited number of elements of the positive class and keeps all the elements of the negative class that just arrived in the current window. The size of BRS is determined by the PIA factor related to the maximum number of iterations of a positive class element that are allowed to remain within the window. In this implementation, SAW performance was evaluated with different values of PIA, as shown in the Figure 13, Figure 14 and Figure 15.

The three figures show that an increase in the value of PIA leads to increased BRS size. It means that more elements of the positive class are retained, so the value of TPR in Figure 13 gradually increased until it reached the highest value of 0.97 when PIA was equal to 12 and then stabilized at approximately the same level. In contrast, TNR decreased as the PIA value increased from 0.99, reaching 0.6 with a value of 15 for PIA.

On the other hand, tracking the performance of the ARF algorithm with SAW using FPR and FNR measures in Figure 13 showed a significant improvement. FPR significantly decreased from 0.99 with a PIA of 1 until it approached zero with a PIA of 15. Furthermore, FNR slowly increased until PIA was 10; after that, the increase was steady. Figure 15 illustrates the outcome of SAW in terms of accuracy and precision, and it confirmed (as in the two previous figures) that the best results were obtained when the value of PIA was between 4 and 9. Although increasing the value of PIA by more than 10 (and thus growing BRS size) led to TPR improvement, it caused more FPR simultaneously. As a result, the accuracy and precision values have declined, with a PIA of more than 10.

The main reason for this behavior is related to the probability of the presence of the Positive and Negative classes after applying SAW. These elements with a high PIA value continued to exist for more time during the model adaptation. The mean probability of a Positive class increased from 0.008 when PIA equals 1 to 0.104 when PIA equals 15. Increasing the PIA value means allowing more similar positive instances to be added into the current window. This increase raises the probability of the positive class and thus decreases the probability of the negative class, thus reducing its dominance. Therefore, the model begins to gradually bias towards the positive class, causing the false positive rate to rise and accuracy to decrease.

However, using PIA as a control parameter controls the size of BRS in a manner that preserves the majority of the negative class. Figure 6 above showed that the probability of a positive class did not exceed 0.08 with a PIA of 8, which means more than 0.92 of the instances of the window stayed in the negative class. On the other hand, following the mechanism of the adaptive sliding window, PIA facilitates forgetting the old positive instances because those instances may not be relevant anymore.

The performance evaluation in this implementation was performed based on the Test then Train method [54]. In this approach, each individual instance in the present window can be utilized for testing the classifier, as it was not seen before. It will then contribute to the training process. The benefit of this approach is that all data instances involve both training and testing, thereby reducing the bias, as the cross-validation method does in batch data classification.

Due to the recentness of the Siena dataset that is used in this evaluation, the number of studies available for comparison is very few. Table 5 summarizes a comparison between the proposed method and two related works. The comparison showed that SAW had better accuracy and TNR. Regarding TPR, it exceeded the study by Sánchez et al. [55], but as SAW tends to preserve a Negative-class majority, it had a lower TPR than Dissanayake et al. [44].

**Figure 16:**
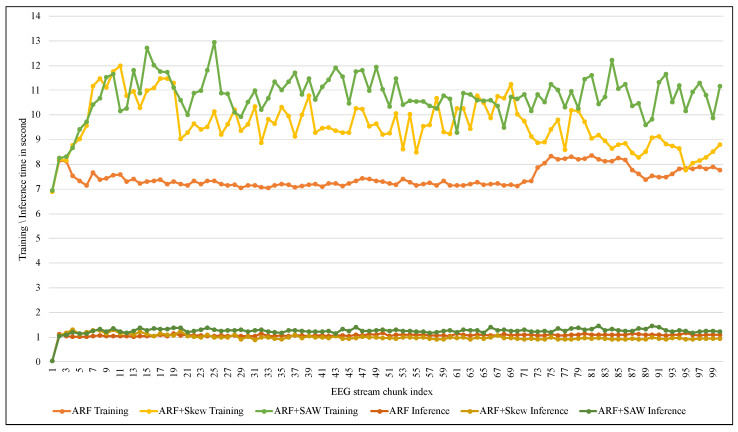
Tracking computational time in seconds.

**Figure 17:**
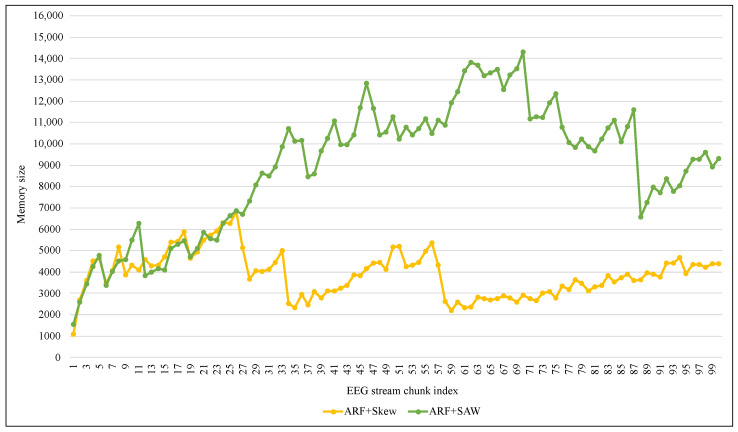
Tracking computational memory cost.

Another comparison has been performed regarding the computational costs, as shown in Table 6, Figure 16 and Figure 17. Regarding the computational time costs, Table 6 and Figure 16 show that SAW needed more training time than other methods due to the calculation-of-distances step in addition to the adaptive size. Although it takes a litte more time in terms of inference time (which is the most important for the epilepsy patient), the average inference of SAW was only 0.74 milliseconds for one instance, as shown in Table 6. In terms of memory consumption, Figure 17 shows that SAW requested more memory resources than Skew. The reason for this difference was the additional size of the SAW adaptive window compared to the fixed size of Skew’s window.

## 5. Discussion

Our results are concerned with the impact of the unbalanced distribution of the data classes of the EEG signals, which are often normal compared to the short time when the epileptic seizure occurred. The above results showed that keeping a specific set of the previous positive class data elements is more effective and reliable than keeping all of them. The essential characteristic of the elements preserved by our proposed method is that they recently arrived, which is consistent with the foundations of data stream mining. In addition, employing the principle of similarity analysis made it possible to determine the data elements closest to the elements in the current window.

Regarding efficiency, although the proposed method adds some steps requiring more considerable computational resources, the main part is calculating the distance between the previous data elements and the current window center. Since the number of these elements is limited, the additional resources required will also be limited. However, this remains a challenge to the proposed method in the case of high-dimensional data. On the other hand, possessing a large number of parameters is one of the most critical determinants of the proposed model, because the ARF algorithm suffers from quite a few parameters that affect its performance, such as the number of basic classifiers. Therefore, adding a new parameter, represented by ATH, to determine the maximum PIA can be challenging in using the proposed model. Thus, the future task of this research is to make the process of determining the value of ATH automatic through several techniques. Since this factor controls the number of elements evaluated for similarity with the center of the current window, methods of controlling the parameters of the clustering algorithms and improving their quality could be used for this task, such as the Elbow Method and Silhouette Method.

The proposed method provides a more reliable classification for determining the onset of an epileptic seizure. It performs this by reducing false alarms associated with an FPR, thus preventing the waste of medical and human resources, and maintaining the patient’s psychological state. On the other hand, the proposed method attempts to reduce the misclassification of the seizure as a normal signal (i.e., TNR) as much as possible because it represents a danger to the patient’s life. Thus, the proposed method can be employed in health institutions and home care for epilepsy patients.

## 6. Conclusions

Similarity analysis plays an important role in many data mining tasks in detecting patterns and clusters. In this work, similarity analysis was utilized to handle the problem of imbalance in the distribution of items in an EEG data stream. The proposed method (SAW) focused on the strength of the presence of the positive class by keeping a certain number of its instances while preserving the majority of the negative class as much as possible. Thus, achieving a balance between the ability to accurately classify the presence of an epileptic seizure or the normal signal on the one hand and reducing false alerts on the other hand. A new and real dataset has been used in the implementation to evaluate the performance of the proposed method. The results showed improvement in the performance of the Adaptive Random Forest algorithm using SAW compared to the Skew ensemble method, in which the False positive rate of ARF was only 0.03 using SAW while it reached 0.81 using the Skew method. On the other hand, F1-score increased from 0.01 to 0.76 using SAW, compared to 0.65 using the Skew method. Finally, the proposed method needs to be improved by selecting the threshold value for the additional positive class set size automatically. 

## Figures and Tables

**Table 1:** The distribution of Normal/Seizure classes in Siena EEG dataset.

Patient ID	File Name	Normal Signals Duration	Seizures Signals Duration	Seizures Signals Ratio
1	PN00-1, PN00-2, PN00-3, PN00-4, PN00-5	11581	315	0.0264
2	PN01-1	12,000	55	0.0045
3	PN03-2	11,581	74	0.0063
4	PN05-2, PN05-3, PN05-4	21727	107	0.0049
5	PN06-1, PN06-4, PN06-5	22662	147	0.0064
6	PN07-1	12,000	63	0.0052
7	PN09-1	8233	81	0.0097
8	PN10-1	9982	70	0.0069
9	PN11-1	8677	56	0.0064
10	PN12-1	9774	64	0.0065
11	PN13-1	9355	49	0.0052
12	PN14-1	7868	28	0.0035
13	PN16-1	8270	124	0.0147
14	PN17-1	9277	71	0.0075
Average	-	11,641.93	93.14	0.0081

**Table 2:** Confusion Matrix of SAW method compared with ARF and ARF + Skew.

Technique \Confusion Matrix		ARF		ARF + Skew		ARF+SAW	
		Predicted Values		Predicted Values		Predicted Values	
		Seizure	Normal	Seizure	Normal	Seizure	Normal
Actual values	Seizure	0	14	668	7	95	7
	Normal	0	1586	750	175	53	1534

**Table 3:** Performance comparison of ARF without data re-balance, with Skew ensemble, and with SAW using the mean values of seven measures.

Technique \Measure	TPR	TNR	FPR	FNR	Accuracy	Precision	F1-Score
ARF	0.0067	0.9998	0.0001	0.9932	0.9912	0.0700	0.0122
ARF + Skew	0.9896	0.1891	0.8108	0.0103	0.5268	0.4710	0.6562
ARF + SAW	0.9313	0.9666	0.0333	0.0686	0.9644	0.6418	0.7600

**Table 4:** Comparison of SAW performance with other three stream classifiers.

Technique \Measure	TPR	TNR	FPR	FNR	Accuracy	Precision	F1-Score
VFDT	0.3981	0.9622	0.0378	0.6019	0.9276	0.5543	0.4633
EFDT	0.3522	0.7751	0.2249	0.6478	0.7487	0.3637	0.3578
KNN	0.7259	0.9515	0.0485	0.2741	0.9379	0.5001	0.5922
ARF	0.9313	0.9666	0.0333	0.0686	0.9644	0.6418	0.7600

**Table 5:** Comparison of SAW method with two research works that used the Sienna dataset.

Research Work\Evaluation Metric	Accuracy	TPR (Sensitivity)	TNR (Specificity)
Dissanayake et al. [44] (2021)	95.88	95.88	96.41
Sánchez et al. [55] (2022)	96	76	-
SAW (the proposed method)	96.44	93.13	96.66

**Table 6:** Comparison of computational time in seconds.

Technique/Time	Window Training	Instance Training	Window Inference	Instance Inference
ARF	7.45588	0.00466	1.06275	0.00066
ARF + Skew	9.61430	0.00601	0.99271	0.00062
ARF + SAW	10.75906	0.00633	1.25258	0.00074

## Data Availability

Publicly available datasets and the source code of ARFC model were analyzed in this study. The files can be found at https://github.com/HayderFatlawi/SAW, accessed on 28 September 2022.

## Abbreviations

The following abbreviations are used in this manuscript:

EEG	Electroencephalography
FFT	Fast Fourier transform
DFT	Discrete Fourier Transform
ADWIN	ADaptive WINdowing
VFDT	Very Fast Decision Tree
EFDT	Extreme Fast Decision Tree
K-NN	K-Nearest Neighbor
SAW	Similarity-based Adaptive Window
BRS	Balance Rare-class Set
PIA	Positive Instance Age
ARF	Adaptive random forest
TNR	True Negative Rate
TPR	True Positive Rate
FPR	False Positive Rate
FNR	False Negative Rate
